# Needing a drink: Rainfall and temperature drive the use of free water by a threatened arboreal folivore

**DOI:** 10.1371/journal.pone.0216964

**Published:** 2019-05-29

**Authors:** Valentina S. A. Mella, Clare McArthur, Mark B. Krockenberger, Robert Frend, Mathew S. Crowther

**Affiliations:** 1 School of Life and Environmental Sciences, The University of Sydney, Sydney, New South Wales, Australia; 2 Sydney School of Veterinary Science, The University of Sydney, Sydney, New South Wales, Australia; 3 Marie Bashir Institute for Emerging Infectious diseases and Biosecurity, The University of Sydney, New South Wales, Australia; College of Agricultural Sciences, UNITED STATES

## Abstract

Arboreal folivores are particularly vulnerable to the impacts of extreme climate change-driven heatwaves and droughts as they rely on leaf moisture to maintain hydration. During these increasingly frequent and intense weather events, leaf water content may not be enough to meet their moisture requirements, potentially leading to large-scale mortality due to dehydration. Water supplementation could be critical for the conservation of these animals. We tested artificial water stations for a threatened arboreal folivore, the koala (*Phascolarctos cinereus*), as a potential mitigation measure during hot and dry weather in New South Wales, Australia. We provided ground and tree drinkers to koalas and investigated changes in use with season, environmental conditions and foliar moisture. Our study provides first evidence of the regular use of free water by koalas. Koalas used supplemented water extensively throughout the year, even during cooler months. Time spent drinking varied with season and depended on days since last rain and temperature. The more days without rain, the more time koalas spent drinking. When temperature was high, visits to water stations were more frequent, indicating that in hot weather koalas need regular access to free water. Our results suggest that future changes in rainfall regimes and temperature in Australia have the potential to critically affect koala populations. Our conclusions can be applied to many other arboreal folivorous mammals worldwide which rely on leaves for their nutritional and water requirements. Artificial water stations may facilitate the resilience of vulnerable arboreal folivores during heat and drought events and may help mitigate the effects of climate change.

## Introduction

Climate change affects the distribution and abundance of animal and plant species by modifying climate regimes, and by causing changes in habitat quality and ecosystem structure [1, 2]. In Australia, a large number of taxa, including corals [3], mammals and birds [4–6], and plants [7] are already impacted by climate change related causes. Trees of the *Eucalyptus* genus, which occupy the majority of Australian forests and woodlands [8], are predicted to undergo significant changes in distribution [9, 10], and in chemical and nutritional leaf composition [11–13] due to climate change. These changes will in turn affect many leaf eating mammals that rely on these plants for their nutritional needs [11, 14, 15]. The koala (*Phascolarctos cinereus*) is one such species.

Koalas are specialist folivorous marsupials native to Australia, which feed almost exclusively on a relatively small number of *Eucalyptus*, *Angophora* and *Corymbia* species [16]. They are widely but patchily distributed over their historical range in eastern Australia, and have been experiencing drastic population declines and local extinctions [17, 18]. They are listed as threatened at both State (Australian Capital Territory, New South Wales and Queensland) and Commonwealth level. The decline of koalas is mostly attributed to habitat loss and fragmentation [19], diseases such as *Chlamydia* [20, 21], and mortality due to fire, attacks by dogs, and vehicle collisions [22, 23]. However, they are also particularly vulnerable to the effects of climate change, suffering heat stress, and because the tree species they rely on are affected by altered temperature and rainfall [24].

Koalas, like other folivores, mostly use dietary water derived from foliage to meet their water requirements [25, 26]. Water content in leaves can be as important as nutrients for koalas and is a key driver of foliage and tree selection [27, 28], especially in hot months [25, 29, 30]. Minimum leaf moisture thresholds required by koalas differ between seasons and are higher in hotter conditions [30, 31], but leaf water is normally sufficient to meet koalas’ moisture requirements [30, 32]. However, during periods of extreme and prolonged high temperatures and dry conditions, leaf water content may not be enough to meet water needs of koalas This often results in physiological stress [33, 34] and large-scale mortality due to dehydration (as seen in [35, 36, 37]). Hence, during heatwaves and droughts, when koalas are limited by the amount of water in the leaves that they eat, free water availability may play an essential role for their survival. However, there is only anecdotal evidence of drinking behaviour in wild koalas [38, 39, 40], and previous literature states that in normal circumstances koalas do not need to drink [25, 41, 42].

Here, we investigate whether koalas would use artificial water stations, whether use varies with seasons, environmental conditions (temperature and rainfall) or foliar moisture, and whether ground or in-tree location for water stations is preferred by koalas. Understanding if koalas access free water is fundamental to determine whether water supplementation can be used as a potential climate change mitigation management tool to improve the resilience of this and other threatened arboreal species during extreme hot and dry conditions.

## Materials and methods

### Study site

The study was conducted on a private property, ‘Dimberoy’ (31°07’33.2”S, 150°00’38.3”E), near the town of Gunnedah, on the Liverpool Plains, in New South Wales, Australia. The Gunnedah region is characterised by a dry, sub-humid climate with a mean annual maximum temperature of 26°C, and mean annual rainfall of 620.4 mm, where winter is usually the driest part of the year [43]. However, the area has recently experienced extended hot and dry summers [43]. In 2006, a state-wide community survey showed that koala populations in the Gunnedah region were the only in the State to show a significant increase in occupancy [44]. However, in 2009 koalas started declining in the area, with many deaths attributed to heatwaves and droughts [35]. The property were the study was conducted is an agricultural farm of about 2100 ha used mostly for grazing cattle. The remnant vegetation consists of open woodland patches of mature *Eucalyptus* trees dominated by poplar box (*E*. *populnea*) and lower numbers of tumble-down redgum (*E*. *dealbata*), white box (*E*. *albens*) and yellow box (*E*. *melliodora*). Koalas in north-west New South Wales live mostly on privately owned agricultural land. Dimberoy has a large free-ranging population of resident koalas.

### Experimental set-up

Ten pairs of water stations were positioned at different sites throughout the property, away from available free water sources (at least 400 m). Sites were at least 500 m apart (based on mean koala home range size at the property, *unpublished data*) to maximise the chances of different koalas visiting the sites and hence ensure independence. Within each site, each water station pair consisted of a ground drinker positioned at the base of a tree, and a tree drinker mounted in the fork of a neighbouring tree at a height of approximately 1.5 m. All trees were heavily used (measured as numbers of koala scats at base of tree) food trees (i.e. eucalypt) and each station pair was set up in trees of the same species.

Each drinker consisted of an automatic refilling drinking bowl (Bainbridge Nylon Automatic Drinking Bowl) with an approximate capacity of 3 L, connected to a water tank (up to 220 L). An adjustable valve maintained a constant level of water in the bowl to provide water *ad libitum*. A ScoutGuard infrared heat-in-motion sensing cameras (model SG560K) was attached 1 m above the drinker facing downwards to record visits (operational hours: 1800–0800 hours) by koalas for a year (March 2016—March 2017). We used JWatcher [45] to quantify *number of visits* and *time spent drinking* (head down, lapping from the bowls at the water stations) by koalas.

We measured air temperature at the trees using Thermochron iButtons (DS1921G; Dallas Semiconductor, Dallas, Texas, USA) attached to the tree trunks at breast height (~1.3 m) on the southwest side of the trees (because this aspect is least exposed to incident solar radiation). The iButtons recorded air temperature data with ± 1 ᵒC accuracy every hour. We obtained maximum daily ambient temperature, mean daily relative humidity and mean daily wind speed from the Gunnedah Airport weather station (www.bom.gov.au). Rainfall quantity (i.e. daily amount of rain and monthly amount of rain), and frequency (i.e. days since last rain) data was obtained from the property rainfall chart records.

### Leaf sampling

To determine seasonal variation in moisture level of different species of eucalypt leaves at the study site, we collected leaves from 53 trees in both cool (September 2016) and hot months (February 2017 and March 2017). Collection of leaves in winter (when rain was most abundant) had to be abandoned due to flooding. Individual trees sampled were restricted to those *Eucalyptus* species selected by koalas for food at the study site [46]: *E*. *populnea* (N = 22), *E*.*dealbata* (N = 10), *E*. *albens* (N = 9) and *E*.*melliodora* (N = 12).

Leaf moisture (% fresh weight) was determined following Ellis et al. [25]. For each tree, we removed about 8 g of mature (fully expanded and developed) leaves from the lower third of the canopy and weighted them to the nearest 0.01 g to determine fresh weight using a digital scale. Water content was determined after drying leaves in a forced air oven at 50°C (to prevent the loss of volatile oils) for 5 days [47]. Water content is represented as (weight loss/fresh weight) x100%.

### Ethical statement

All specific permissions required for the activities conducted in the study, including sampling procedures and experimental manipulations, and permission to work with threatened species, were approved by the University of Sydney Animal Ethics Committee (#2016/955) and conducted under the NSW National Parks and Wildlife Service Scientific License SL101687. The land owner gave permission to conduct the study on his property.

### Statistical analysis

We used the proc glimmix procedure in SAS (SAS 9.4) to test if *position* of the water stations (ground or tree) and *season* had an overall effect on *number of visits* and *time spent drinking per visit* in separate models. We then tested the effect of climate variables on *time spent drinking per visit* by running another model including factors related to rainfall and temperature (i.e. *days since last rain*, *tree temperature* and *wind*). We used a generalized linear mixed effects model (GLMM) with a poisson distribution and loglink function for count (visit) data, and GLMMs with a lognormal distribution and identity link function for behavioural data. *Site* and *drinker* (nested within site) were the random factors in all three models. We used Tukey-Kramer tests for post-hoc comparisons.

Climatic pairs of variables with a Pearson coefficients > ± 0.5 were considered proxies of one another and only one was included in our third model [48]. We chose to use *days since last rain* (and not other variables related to quantity of rain) to account for the lag effect of rain [34] and because rainfall is patchy in the Liverpool Plains region (BOM 2018). We used *temperature at the tree* (and not *maximum daily ambient temperature*) because one of the strategy used by koalas to thermoregulate is to select cooler trees when temperature is high [49] and dissipate heat by hugging the tree trunks [50, 51].

Finally, we used a GLMM with gaussian distribution and identity link function to test whether *leaf moisture* at the study site varied between *tree species*, *season* and with the interaction *tree species* x *season*. *Tree ID* was included in the model as a random factor.

## Results

**Fig 1:**
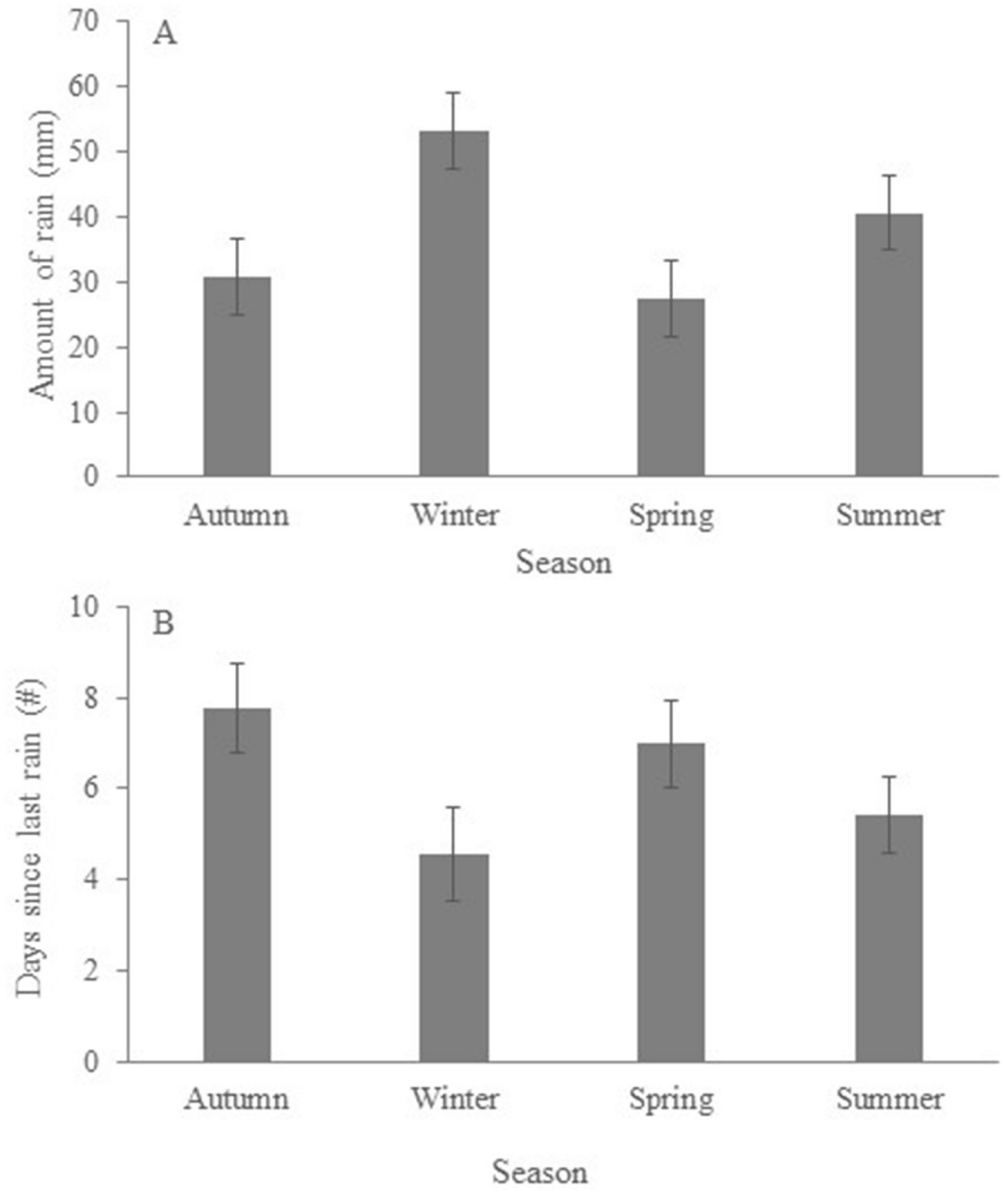
Mean ± SE seasonal rainfall obtained from the Dimberoy weather station between March 2016 and March 2017; (a) quantity (i.e. amount of rain) and (b) frequency (i.e. days since last rain).

**Fig 2:**
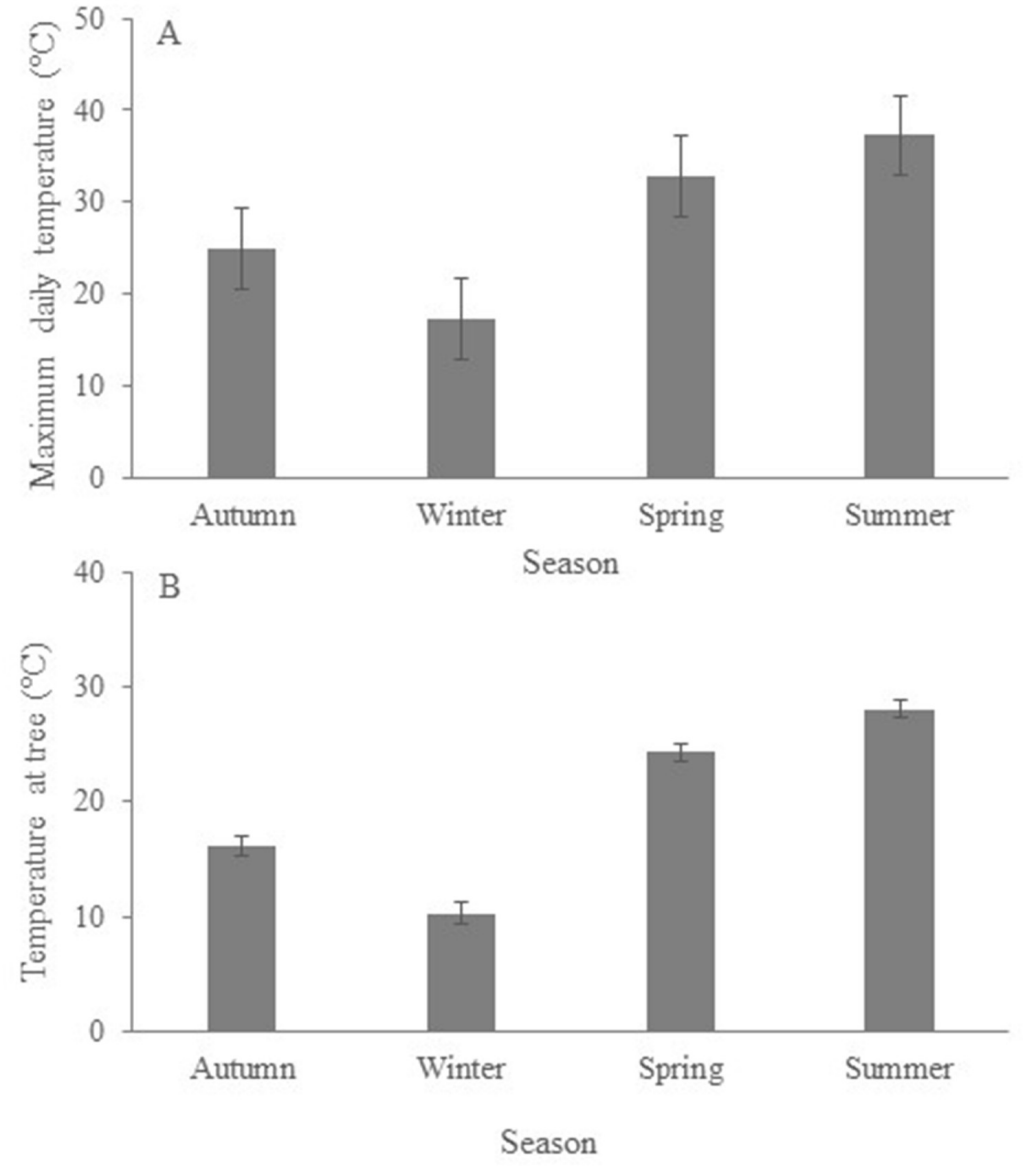
Seasonal variation in temperature; mean (± SE) (a) maximum daily temperature (source: BOM 2018) and (b) mean (± SE) air temperature (measured with iButtons) at koala food trees at the study site.

Mean annual (March 2016—March 2017) rainfall during our study was 637 mm, with rain more abundant in winter than in any other season (Fig 1a) but less frequent than in autumn and spring (Fig 1b). Mean annual maximum temperature was 26.2°C (BOM 2018). However, summer maximum daily temperatures (Fig 2a) were often above 40°C (highest daily temperature recorded was 45.6°C in February 2017) and summer night-time temperatures frequently exceeded 30°C (BOM 2018). *Temperature at the tree* (Fig 2b) was highly correlated with *maximum daily temperature* (r = 0.97; P < 0.0001) but was always lower.

### Behaviours at water stations

**Fig 3:**
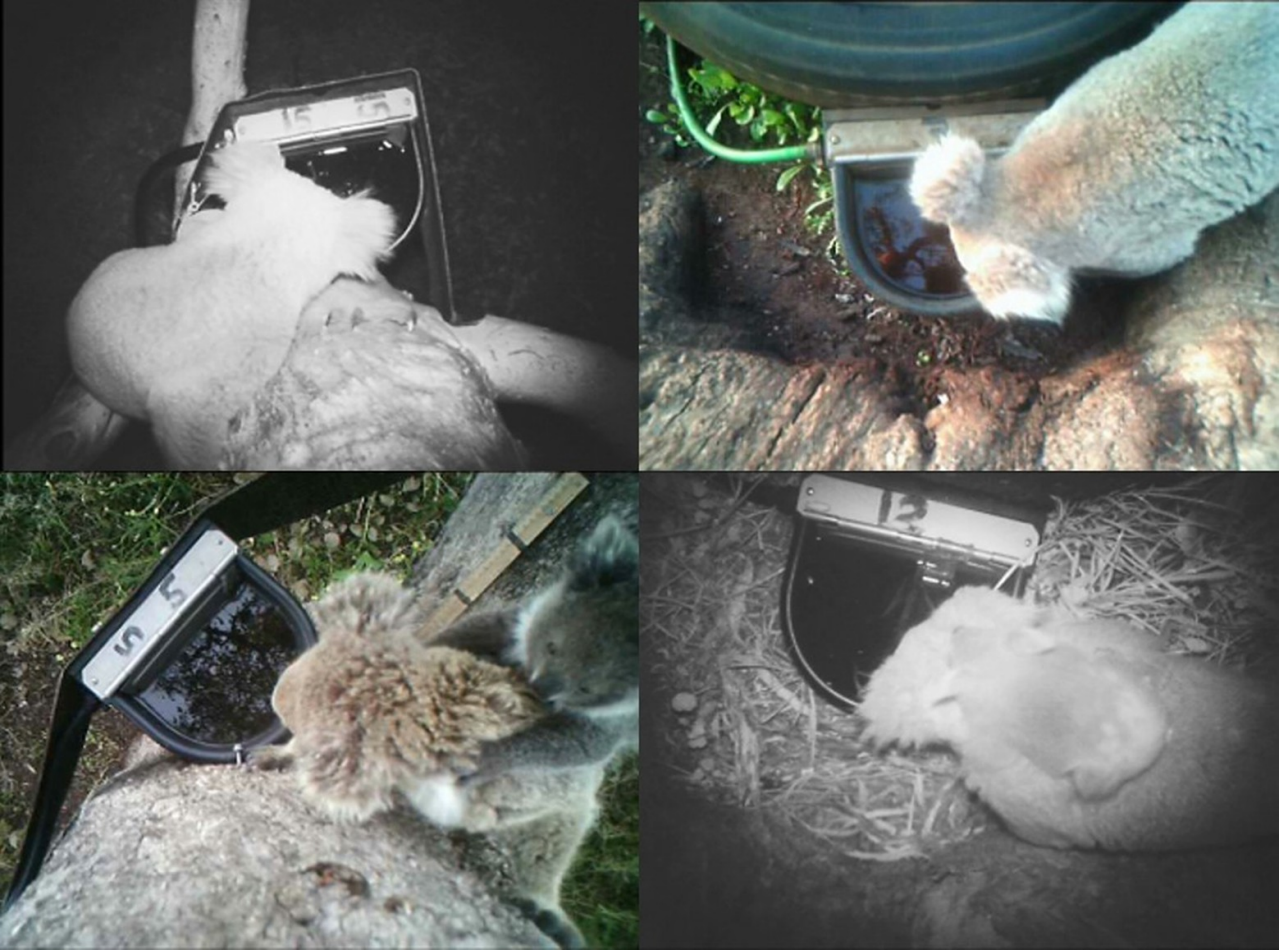
Koalas drinking from tree (left) and ground (right) artificially supplemented water stations at Dimberoy.

All water stations were used extensively by koalas throughout the year (605 visits in total). Of these, 401 visits resulted in koalas drinking. Koalas always visited the drinkers alone, except for females carrying a young (Fig 3) and the latter never drank. Koalas spent 56% of the total time at the water stations engaged in *drinking* behaviour. The rest of the time was spent *moving* (16%), *vigilant* (9%), *investigating* the stations (5%) and in *other* less frequent behaviours such as self-grooming, scratching and calling for mates (14%).

Numerous other animals visited the drinkers, including sugar gliders (*Petaurus breviceps*), feathertail gliders (*Acrobates pygmaeus*), brushtail possums (*Trichosurus vulpecula*) at tree drinkers and echidnas (*Tachyglossus aculeatus*), Eastern grey kangaroos (*Macropus giganteous*), hares (*Lepus europaeus*), feral cats (*Felis catus*) and red foxes (*Vulpes vulpes*) at ground drinkers, though the latter was also detected in trees [52].

### Seasonal effect on visits

**Fig 4:**
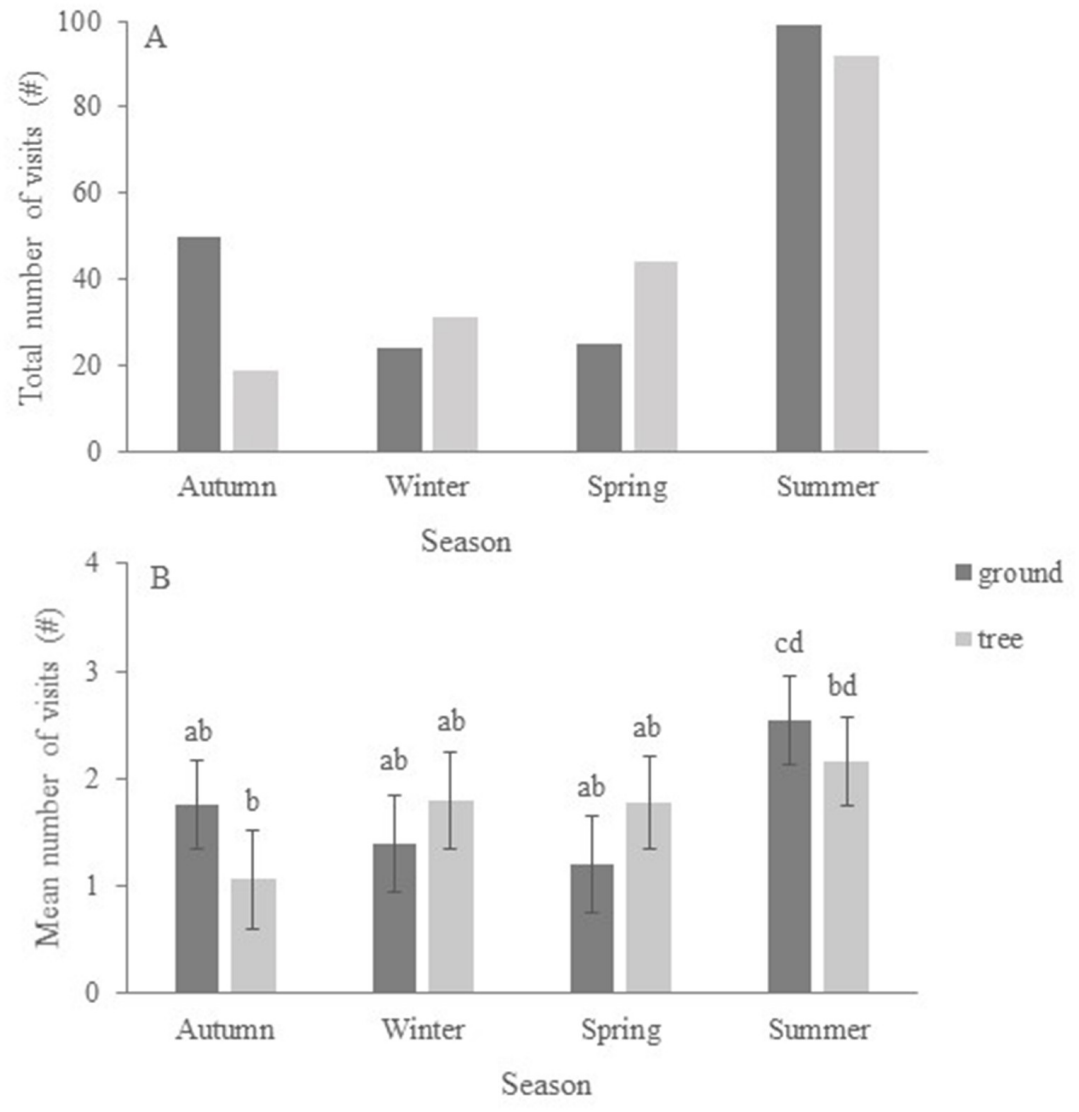
Seasonal visits to water stations by koalas; (a) total number of visits (cumulative) and (b) mean number of visits to ground and tree drinkers. Values are Least Squares Means (± SE). Different letters indicate significant differences.

*Number of visits* by koalas depended on *season* (F_3,11_ = 20.12; P < 0.0001) but not on *position* of the drinker (F_1,11_ = 0.02; P = 0.968), though there was a significant effect of the interaction *position* x *season* (F_3,11_ = 6.18; P = 0.010). In summer, koalas visited the drinkers more (total number of visits) than in any other season (Fig 4a) but mean number of visits to ground and tree drinkers varied depending on the season (Fig 4b).

**Fig 5:**
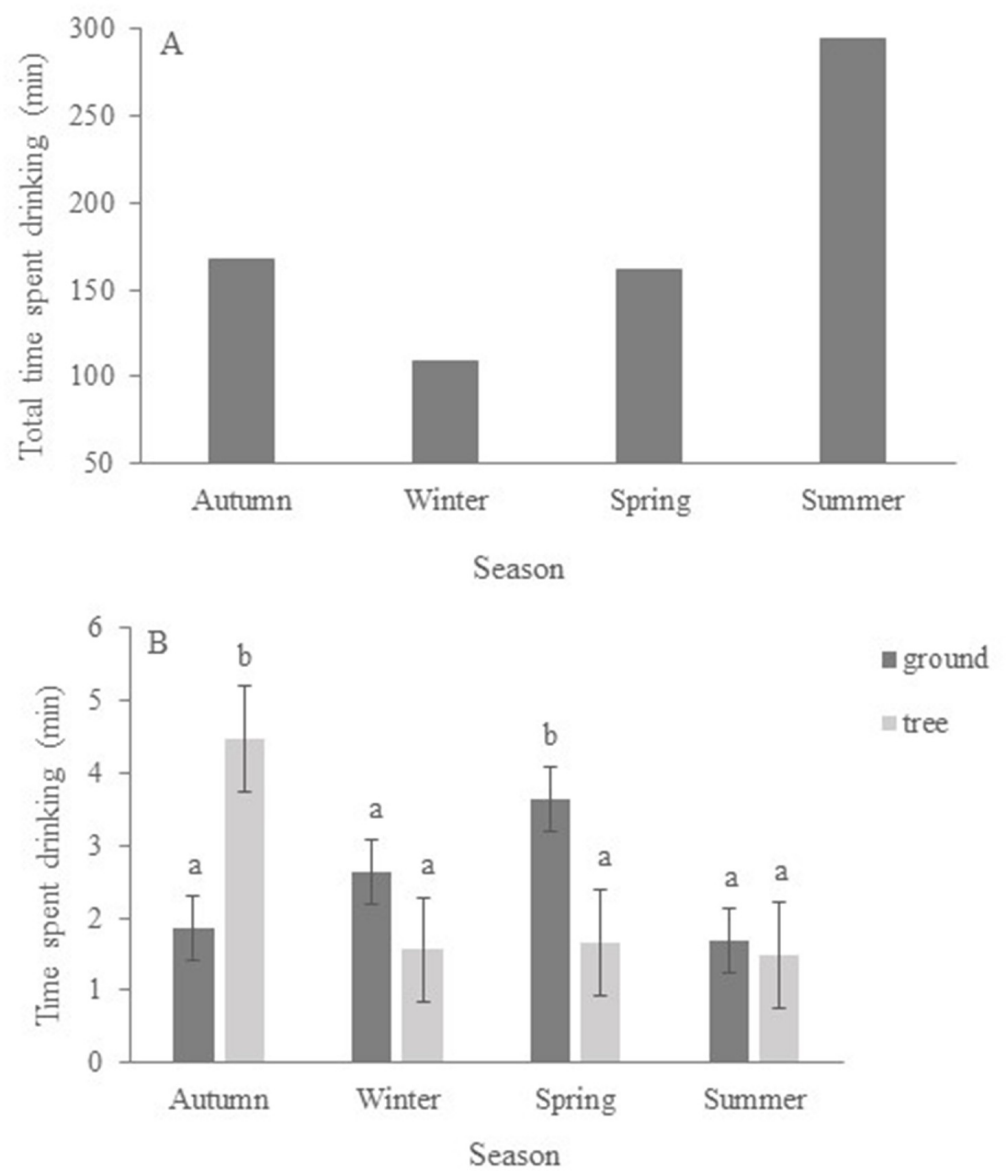
Time spent drinking by koalas in different seasons; (a) total time (cumulative) and (b) mean time per visit at ground and tree drinkers. Values are Least Squares Means (± SE). Different letters indicate significant differences.

*Time spent drinking per visit* by koalas depended on *season* (F_3,349_ = 4.70; P = 0.0031). There was no overall effect of *position* of the drinker (F_1,349_ = 0.18; P = 0.671) but there was a significant *position* x *season* interaction (F_3,349_ = 6.85; P = 0.0002). Total time spent drinking (cumulative) was greater in summer than in other seasons (Fig 5a) but koalas’ preference for drinkers’ position varied with season (Fig 5b).

### Weather effect on visits

*Time spent drinking per visit* by koalas depended on *days since last rain* (F_1,344_ = 7.80; P = 0.005) and *temperature at the tree* (F_1,344_ = 4.95; P = 0.027) but not on *position* of the drinker (F_1,344_ = 0.44; P = 0.506) and *wind* (F_1,344_ = 0.49; P = 0.484). Koalas spent more time drinking per visit when rain was infrequent and when temperature at the tree was lower (Table 1).

**Table 1:** Generalized linear mixed effects model testing the effect of weather variables (wind, temperature and rainfall) and position of the drinkers on time koalas spent drinking per visit. Asterisk (*) indicates significance.

Variable	Estimate	SE	df	t	P
Intercept	0.444	0.297	5	1.49	0.195
Position of the drinker	-0.125	0.187	344	-0.67	0.506
Wind	-0.009	0.012	344	-0.70	0.485
Temperature at tree	-0.020	0.009	344	-2.22	0.027*
Days since last rain	0.038	0.013	344	2.79	0.006*

### Leaf moisture

Mean leaf water content at the study site was 53% fresh weight and did not change with *season* (F_1,68_ = 2.14; P = 0.148), did not vary significantly between *species* (F_3,68_ = 1.56; P = 0.208; *E*. *albens* 52%, *E*. *dealbata* 52%, *E*. *melliodora* 54%, *E*. *populnea* 53%), and was not influenced by the interaction *season* x *species* (F_3,68_ = 2.13; P = 0.104).

## Discussion

Our study is the first to document the use of free water by koalas, suggesting that water supplementation may help this arboreal folivore during extreme weather events. We show that koalas visit artificial water stations extensively and that the use of free water depends on rainfall and temperature. Water stations are used more in hot and dry periods, suggesting that free water may be needed by koalas in extreme climatic conditions.

Koalas showed no clear preference for ground or tree drinkers and used both throughout the year. However, number of visits and time spent drinking at ground and tree drinkers varied with season. For example, in spring koalas spent more time drinking at ground water stations than at those in trees, while in autumn the pattern was reversed (Fig 5b). This might be because koalas prefer to rest in different position in the trees across seasons. In warmer weather, koalas can often be found at the base of trees to escape the heat [53], while in cooler conditions they spend more time in the higher tree canopy [50]. Hence, koalas might have accessed drinkers opportunistically and selected the most convenient position (ground or tree) depending on seasonal encounter rate.

Water stations also attracted other herbivores and carnivores, including feral animals like hares, cats and foxes. To reduce the likelihood of these species gaining access to water and to minimise the risk of predation on the ground, we suggest providing water above ground in future supplementation studies targeting arboreal species such as koalas.

In summer, when temperature was high and water loss more acute and rapid than in other seasons, the use of both tree and ground drinkers increased. Total number of visits (Fig 4a) and total time spent drinking (Fig 5a) doubled compared to other seasons. However, time spent drinking per visit (Fig 5b) was comparatively greater in spring (at ground drinkers) and in autumn (at tree drinkers) than in summer (at either locations). This is because koalas employed different behavioural strategies to obtain water depending on temperature. In summer, when temperature was extreme, they drank for a shorter duration at each visit but visited more frequently, while in autumn and in spring, they had comparatively longer drinking sessions but visited water stations less often (Figs 4 and 5).

Frequent access to free water may be fundamental for koalas to assist thermoregulation when temperatures are high. Koalas reduce evaporative cooling from the respiratory tract, which accounts for their greatest water loss [54], by using tree trunks to dissipate heat and thermoregulate [50]. However, the hotter the temperature, the harder would be for koalas to achieve adequate conductive heat loss; and the more water koalas lose through evaporative cooling, the more water they will need to meet water requirements. Hence, the capacity of koalas to thermoregulate in extreme heat might depend exclusively on the availability of water (free or as leaf moisture) [32].

The longer between rain events, the more time koalas spent drinking per visit. This may suggest that when rain is scarce, leaf moisture might not always be sufficient to meet water needs. *Eucalyptus* foliage normally contains over 50% water but this depends on water availability to the tree (rainfall and surface water) [28] and koalas prefer leaves with high moisture content [33]. The mean percentage of water in leaves in our study (53% fresh weight) was below the 55–65% preference level identified for koalas, under which foliage (including the four *Eucalyptus* species sampled here) is rejected in captivity [29, 30]. Koala deaths have been reported in Queensland when leaf moisture fell below 51% fresh weight [31], indicating that there might be minimum leaf water threshold levels required for koalas to sustain their water requirements and these will likely vary with climatic conditions. Leaf moisture in our study, although not extremely low, did not appear to adequately meet these requirements when temperature was high and rain was infrequent, as water stations were used extensively by koalas.

Leaf moisture did not vary between species nor did it change between cool and hot months. It is possible that we have missed subtle changes in leaf moisture as this was only measured three times in a year. However, we consider this unlikely as other studies have found little seasonal variation in leaf moisture over one year [55] and a lag effect (up to six months) of rainfall on leaf water content [56]. Nevertheless, in future studies, we recommend measuring foliar moisture more often throughout the year to ensure that finer changes (if existent) can be detected.

Many koalas at the study site are affected by *Chlamydia pecorum* [57], a bacterial infection that causes urinary and reproductive disease in koalas [58]. It is unknown whether the disease influences their drinking behaviour but koalas have been observed drinking profusely when unwell [59]. We could not identify individual koalas at drinkers, hence, it was not possible to link drinking behaviour to disease status. In future studies, it would be beneficial to determine koalas’ identity so the effect of disease on drinking behaviour can be tested.

Our findings suggest that when temperature is high and rain scarce, koalas intensify their drinking behaviour to sustain their increased water requirements as a strategy to cope with extreme weather conditions. Heatwaves and droughts are becoming increasingly common and severe in Australia [60] and climatic warming is predicted to have severe effects on plants [61] and fauna [62], especially in eastern Australia, where koalas are distributed [16]. Our results support previous research suggesting that rising temperatures and lack of rainfall likely play a pivotal role in koalas’ decline [63, 64].

Similar results may be expected for many arboreal folivorous animals which rely on leaves for their nutritional and water requirements. For example, the distribution of another specialist tree-dwelling folivore, the green ringtail possum (*Pseudochirops archeri*), is limited by the duration and severity of extreme weather events and the availability of free water and leaf moisture, with range contractions, population declines and extinctions predicted with climate change [65]. Similarly, climatic changes such as drier and prolonged dry seasons, limit reproduction and survival of Milne-Edwards' sifaka (*Propithecus edwardsi*), a folivore lemur from Madagascar [66]. Hence, climate change could reduce the capacity of many folivores to persist in their original distribution range.

Based on our results, one mechanism for these contractions is the increased need of water to sustain water requirements. Arboreal folivorous mammals like the koala, are limited in their food intake by leaf toxins [30, 67], which in turn affect their water intake in the form of foliar moisture. Hence, they simply cannot eat more leaves if they need more water. Increased CO_2_ emissions are predicted to increase the level of phenolics and tannins in plants [68, 69], including in *Eucalyptus* leaves [70], further reducing the capacity of koalas to gain enough foliar water. Therefore, supplementing free water might represent one of the ways to reduce the detrimental effects on koala’s food trees expected with climate change.

### Conclusions

We have shown that koalas use supplemented water extensively throughout the year and in particular if temperature is high or rain infrequent. Our results overturn previous understanding of how koalas stay hydrated in the face of a changing climate. Artificial water stations have been identified as a powerful tool for maintaining biodiversity in arid zones [71] including in Australia [72], although they may also attract and favour feral species [52]. Our study demonstrates that they may also represent a practical management tool for the conservation of vulnerable arboreal folivores experiencing water deficient conditions. Provision of supplementary water sources may be identified as a basic need to help these animals survive through long dry and hot spells, when ground water is non-existent or when rainfall events are widely separated, but benefits have to be considered against costs related to non-target species. The next step is quantification of the potential benefit of water stations as a mitigation tool against heat-stress during heatwaves and droughts. Longer-term monitoring is required to determine if water supplementation can maintain populations throughout periods of extreme weather. Future studies should include measurement of water turnover rates and explore how free water availability affect animal’s health.

## Supporting information

S1 Dataset Koala drinking behaviour data. All data used in the study are available as supporting information.
(XLSX)
